# War, Psychology and Eugenics

**Published:** 1914-09

**Authors:** 


### War, Psychology and Eugenics.

Racially speaking, war should be stigmatized as a crime. And the present European carnage as the most atrocious perpetrated in the annals of history. There may be principles which any nation must defend. There may be questions of honor which a recourse to arms alone can satisfy. But the wanton destruction of human life, involving as it does the highest potential factors of a race, is a crime of momentous consequences. Society represents the accumulation of cultural advancement which is its stock in trade for the development of the next generation. To destroy this resource; to lose this aggregate of inherent qualities acquired at such a cost of time and struggle is so prodigious, so amazing that it staggers the most vivid imagination. And then, when it dawns upon us that the highest civilization yet attained in the world is imperiled with complete destruction the hope of the eugenist and the dream of the reformer is utterly shattered. The crime is all the greater because this is true.

A country's boasted conquest of war it not a glorious heritage. For it is never gained except at the cost of irretrievable racial factors. It is not the weak and the invalid, not the inferior or hopeless classes that are killed in war, but it is always the virile and strong, the best heredity, the highest specialized talent that is sacrificed. This is the crime, the penalty follows. Out of the ashes and ruins of this human massacre will rise the remaining signs of life, ill-fitted to struggle against the consequences following in the wake of war—the most inferior inherent qualities to replenish the next generation.

But as incredible as these facts may seem the cause is not difficult to understand. Viewed from the eugenist standpoint it was inevitable. It is simply a nation gone mad, a case of monomania. In place of its being an individual, it is a mob. There is a limit to human endurance. Let this be overtaxed and there is a breaking point. When the mental poise is gone there can be no control. These nations for forty years have been harboring resentment and retaliation against one another until fear has become an obsession. They have been dreaming of conquests, thinking war, mimicking war and preparing for war until the psychological limit has been reached. What force could stem such a tide?

Will we ever learn that there is a mental as well as a physical hygiene. That mental overstrain and negative mental influence will result in mental unbalance and inefficiency the same as the abuse of physical law will cause deterioration and weakness? Will our country profit by the costly lesson taught by time and the experience of this present conflict? Or, after all, will we too be swept on in the tide of human destiny without choice or power to control our fate? Will a more enlightened race avoid pitfalls that harbor destruction and purposely cultivate those conditions which alone can endure?

